# Fungal BGCs for Production of Secondary Metabolites: Main Types, Central Roles in Strain Improvement, and Regulation According to the Piano Principle

**DOI:** 10.3390/ijms241311184

**Published:** 2023-07-06

**Authors:** Alexander A. Zhgun

**Affiliations:** Group of Fungal Genetic Engineering, Federal Research Center “Fundamentals of Biotechnology”, Russian Academy of Sciences, Leninsky Prosp. 33-2, 119071 Moscow, Russia; zzhgun@mail.ru; Tel.: +7-916-974-9769

**Keywords:** secondary metabolites, biosynthetic gene clusters (BGCs), filamentous fungi, global regulation, LaeA

## Abstract

Filamentous fungi are one of the most important producers of secondary metabolites. Some of them can have a toxic effect on the human body, leading to diseases. On the other hand, they are widely used as pharmaceutically significant drugs, such as antibiotics, statins, and immunosuppressants. A single fungus species in response to various signals can produce 100 or more secondary metabolites. Such signaling is possible due to the coordinated regulation of several dozen biosynthetic gene clusters (BGCs), which are mosaically localized in different regions of fungal chromosomes. Their regulation includes several levels, from pathway-specific regulators, whose genes are localized inside BGCs, to global regulators of the cell (taking into account changes in pH, carbon consumption, etc.) and global regulators of secondary metabolism (affecting epigenetic changes driven by velvet family proteins, LaeA, etc.). In addition, various low-molecular-weight substances can have a mediating effect on such regulatory processes. This review is devoted to a critical analysis of the available data on the “turning on” and “off” of the biosynthesis of secondary metabolites in response to signals in filamentous fungi. To describe the ongoing processes, the model of “piano regulation” is proposed, whereby pressing a certain key (signal) leads to the extraction of a certain sound from the “musical instrument of the fungus cell”, which is expressed in the production of a specific secondary metabolite.

## 1. Introduction

The production of secondary metabolites (SMs) is one of the most prominent biochemical attributes of filamentous fungi (or moldy fungi, or molds), and has stimulated extensive research on these microorganisms since the 1950s [1,2,3]. As a result of this, numerous compounds have been discovered, some of which are capable of harming human health, while others are able to heal people [4,5,6,7]. In parallel with the emergence of new knowledge about the effects of these low-molecular-weight compounds on the human body and the deciphering of their structures, investigations have been carried out that aim to study the mechanism of action at the cellular level [8]. For example, targets for the main classes of antibiotics have been identified, and the mechanisms for the emergence of resistance in microorganisms against these drugs have been established [9,10,11,12,13,14]. In the late 1980s, in light of the emergence of the era of genetic engineering, the molecular basis of the biosynthesis of secondary metabolites began to be studied [15]. In particular, the so-called biosynthetic gene clusters (BGCs) responsible for the biosynthesis of the corresponding SMs were discovered [16,17]. This knowledge made it possible to apply the strategy of reverse genetics, i.e., going from gene to trait/phenotype, and search for an appropriate product for “silent” or so-called “orphan” BGCs [18,19,20]. Currently, there are various techniques for such genome-mining of BGCs [15,21,22,23,24,25]. Emerging knowledge about biosynthetic gene clusters for the production of secondary metabolites, as well as a lot of difficulties associated with the “awakening” of silent BGCs, have led to the understanding, in numerous studies, of the existence of a complex regulatory system for them [26,27,28,29]. Such regulation operates in concert at several levels, starting with cluster-specific regulators, transcription factors whose genes cluster within a particular BGC and regulate the expression of the same BGC, ending with global regulators and chromatin-mediated regulation [30,31,32,33]. Due to the presence of such a system of regulation, there is a relationship between the production of SMs and the development of fungi [34]. The production of the corresponding SMs occurs at certain stages of the development of the fungus; for example, the synthesis of pigments occurs after the transition from the growth phase (trophophase) to the production phase (idiophase) [35]. On the other hand, most fungal BGCs are silent under normal physiological conditions and begin to work after receiving an appropriate environmental signal that affects the regulatory system [36,37,38]. Composite pleiotropic events accompanying the functioning of the fungal secondary metabolism are currently being studied using complex, including multi-omics, approaches [39,40,41].

The existing fundamental knowledge of the biosynthesis and regulation of SMs in filamentous fungi is extremely important, since, based on natural isolates, over the past 70–80 years, numerous industrial producers of pharmaceutically significant drugs, such as antibiotics, statins, and immunosuppressants, have been created [2,42,43,44]. Numerous works are also underway to create strains-producers of antitumor drugs that are synthesized in fungi [45]. Such industrial producers have been obtained as a result of the so-called classical strain improvement (CSI) methods associated with random mutagenesis and screening for the production of targeted SMs [46,47]. Modern knowledge about the organization of the regulatory machinery of secondary metabolism in the fungal cell makes it possible to understand the molecular basis of the direction of mutational selection, leading to high-yield production of the target secondary metabolite [48]. To achieve this, the original wild-type strains and improved producers are compared at the reference points of improvement programs [49]. Understanding the changes that have taken place is important for the development of future approaches to the targeted genetic engineering of high-yielding fungal producers of SMs [50,51].

Numerous reviews published by highly qualified researchers on the topic of secondary metabolites have taken their rightful place in the classification [1,5,15,30,40,52,53,54,55,56]. However, it is becoming increasingly difficult to grasp such knowledge from various points of view, starting with the structures of molecules, the organization of biosynthetic gene clusters, and their regulation at various levels. And this gap between the volume of formal numerical volumes of knowledge and the possibility of their perception by a person will increase if they are not classified or ordered (packed) in a model. In this review, an attempt is made to communicate knowledge related to the functioning and regulation of secondary metabolism in filamentous fungi, as well as that related to the key changes that occur when they are improved via classical methods [48,49,57,58,59]. For a more visual perception of these processes, the model “regulation of the secondary metabolism of filamentous fungi according to the piano principle” is proposed. This model aims to simplify the understanding of the numerous and complex processes of the signaling and regulation of the biosynthesis of SMs in filamentous fungi and their changes in high-yielding industrial producers. To achieve this, at the associative level, the processes occurring in the fungal cell after the corresponding signal and leading to the production of one secondary metabolite or another are compared with the impact, in which the corresponding sound is extracted after pressing a specific piano key.

## 2. Main Types of Fungal Secondary Metabolites

**Figure 1:**
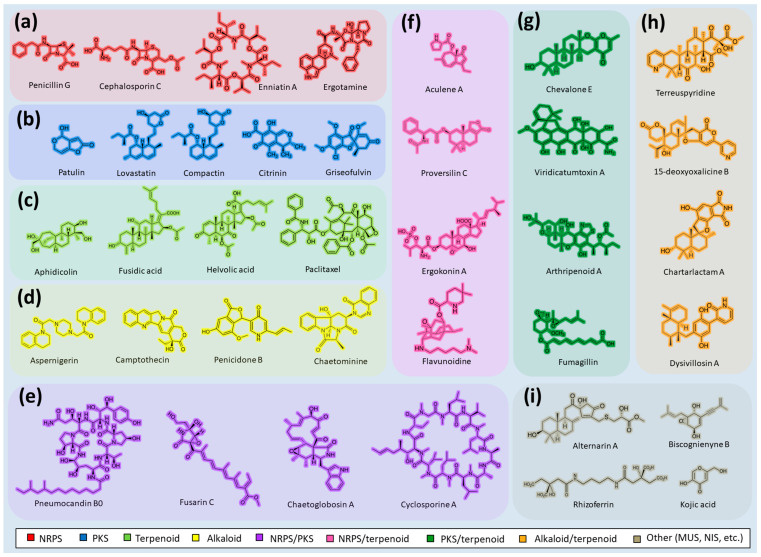
Chemical structures of the main types of secondary metabolites (SMs) produced by filamentous fungi, based on the enzymatic activity of: (**a**) nonribosomal peptide synthetase (NRPS); (**b**) polyketide synthase (PKS); (**c**) terpene cyclase (TPC) for terpenoid production; (**d**) a number of enzymes for alkaloid production; (**e**) NRPS and PKS for production of NRPS/PKS hybrid; (**f**) NRPS and TPC for production of NRPS/terpenoid; (**g**) PKS and TPC for production of PKS/terpenoid; (**h**) enzymes for alkaloid production and TPC for production of alkaloid/terpenoid; (**i**) other enzymes for production meroterpenoid with unique structures (MUS), or NRPS-independent siderophore (NIS), or other types of molecules.

Fungi are one of the most evolutionarily adapted organisms, which has allowed them to occupy the majority of ecological niches suitable for existence on Earth over the past billion years [60,61]. According to existing estimates, global fungal diversity is about an order of magnitude greater than that of land plants [62,63]. One of the paramount assistants to such adaptive expansion was the ability to produce wide-variable low-molecular compounds, the so-called secondary metabolites, in response to changes in the state of both the organism itself and the environment [64,65,66]. These highly active molecules have begun to play a trigger function, and are selected as keys to the locks of various processes in the development of the organism itself, and its defense and/or attack against surrounding organisms and other species via within- and between-species interaction. More than 15,000 biologically active SMs are currently known to be produced by fungi (which is approximately 50% of all known biologically active SMs from microorganisms), some of which are used in pharmaceutical, agrochemical, and cosmetic products [52]. The majority of these compounds belong to one of four classes obtained through the activity of: (i) nonribosomal peptide synthetase (NRPS), (ii) polyketide synthase (PKS), (iii) terpene cyclase (TC) for terpenoid production, or (iv) a number of enzymes for alkaloid production (Figure 1).

There are also a number of hybrid variants of fungal SMs, which are obtained due to combinations of the main four biosynthetic strategies, for example, the NRPS/PKS hybrid, or meroterpenoids, such as NRPS/terpenoid, PKS/terpenoid, and alkaloid/terpenoid [67]. Finally, SMs of fungi are known that do not belong to any of the four major types, or their hybrid derivatives, for example, the NRPS-independent siderophore (NIS) [68]. 

Typically, the molar mass of fungal SMs ranges from 140 to 1200 or more, with the vast majority ranging from 250 to 600 (Table 1). Perhaps it is precisely these molecular sizes that make it possible to create, on the one hand, a huge variety of chemical structures (based, for the most part, on the atoms of H, C, O, N, P, and S), which, on the other hand, can serve as small keys to the locks of macromolecular structures. These keys are uniquely sharpened for a specific task, for opening a particular lock, which must be unlocked at a strictly specific moment. More detailed information on the characteristics of representatives of the main types of fungi secondary metabolites is presented in Table 1.

## 3. Biosynthesis of Fungal Secondary Metabolites in Response to Signals

**Figure 2:**
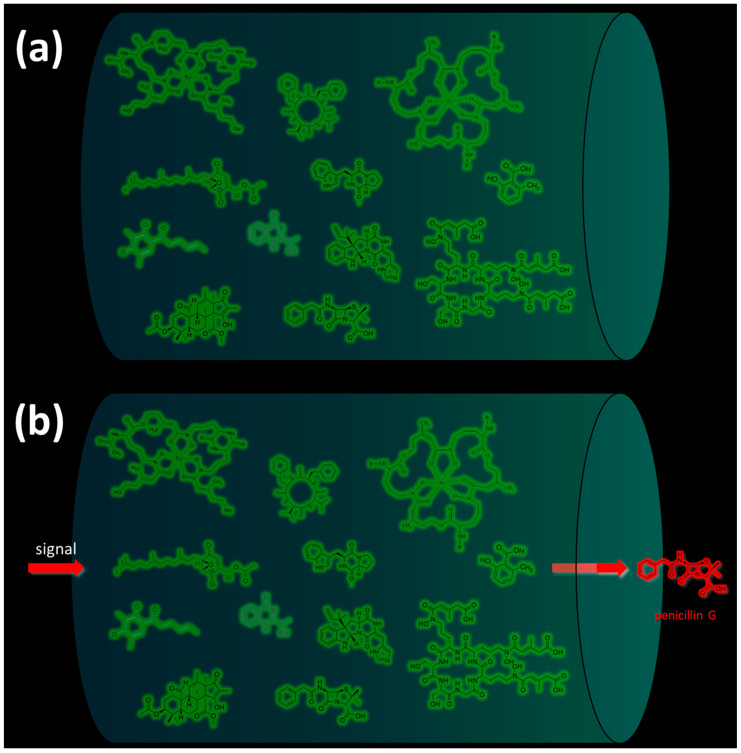
Biosynthesis of secondary metabolites (SMs) in response to signal exposure. The arrival of a specific signal (from the external environment or the internal signal of the cell) leads to the production of corresponding SMs. As an example, changes in the production of SMs in *Penicillium chrysogenum* are given: (**a**) Under normal physiological conditions (in the absence of specific environmental signals) and at an early stage of fungal cell development (trophophase stage), most SMs are not produced. (**b**) In response to a specific signal, the corresponding SM is synthesized. The green color shows known SMs of *P. chrysogenum*, which, in principle, can be synthesized by the cell (representing its biosynthetic capacity), but are not produced at a particular moment. The red color shows the currently produced SMs in response to the signal; the antibiotic penicillin G, synthesized in response to an external signal, is given as an example.

In most cases, under normal physiological conditions during the trophophase, fungal SMs are not synthesized (Figure 2a) [35]. However, under the influence of certain internal or external signals, cellular mechanisms are triggered, leading to the synthesis of one corresponding (target) SM or another (Figure 2b) [1,15,134].

Low-molecular-weight compounds, including SMs, are one of the main methods of communication between microorganisms [135,136,137]. If a civilized person uses several thousand words for everyday communication, then microorganisms “speak” the language of several hundred low-molecular-weight compounds [138,139]. Thus, in the composition of the microbiome, individual species can “carry on new conversations”, producing SMs that are not detected in the composition of a monoculture [140].

The SMs of microorganisms play a significant ecological role [36,141]. They can be used as weapons and armor in cases of a confrontation between microorganisms [142]. On the other hand, the SMs of fungi can serve as important agents at the stages of infection in plant and animal cells [143,144,145,146,147,148]. Furthermore, fungal SMs can serve as communication molecules [149,150,151], playing a significant role in the fungal “communicome” [152,153]. Fungi use other low-molecular-weight molecules than bacteria for quorum sensing, such as tyrosol, farnesol, and butyrolactone-I [149,154]. Along with this, fungal SMs can inhibit the quorum sensing systems of competing microorganisms [149,155].

In response to low levels of iron in the environment, fungi synthesize siderophores, special compounds with a high affinity for iron ions [156]. They are secreted into the external environment to chelate trace amounts of iron; the resulting complexes of siderophores with iron have an increased affinity for special cellular receptors, as a result of which the necessary iron enters the cell [157]. The synthesis of siderophores is also important in the pathogenesis of a number of fungi [147].

Fungal SMs are capable of manipulating plant community (plant microbiome) dynamics by inhibiting or facilitating the establishment of co-habituating organisms and mediating fungal–bacterial, fungal–fungal, and fungal–animal interactions associated with the plant community [158]. The production of SMs in fungi is influenced by environmental factors; for example, their production in fungi that have lived for hundreds and thousands of years in lichens is affected by light, UV radiation, altitude, temperature fluctuations, and seasonality [159].

## 4. Biosynthetic Gene Clusters (BGCs) for the Production of Fungal Secondary Metabolites

One of the revolutionary discoveries in understanding the molecular basis of the biosynthesis of SMs was the identification of so-called biosynthetic gene clusters (BGCs) [160,161,162,163]. It turned out that in order to create a particular natural product, microorganisms and plants have an appropriate set of genes that are in relative proximity in a particular region of the chromosome (clustered) and are jointly regulated [164]. Thus, the genes responsible for the stages of biosynthesis of a particular SM are either “silent” together or jointly upregulated [26]. The architecture of metabolism itself leads to the maximization of biosynthetic diversity in fungi [165]. For example, a number of BGCs have biased ecological distributions, consistent with niche-specific selection [165]. Several thousand BGCs are currently known in fungi; it is assumed that their numbers range from 100,000 to millions [1,160,166].

**Figure 3:**
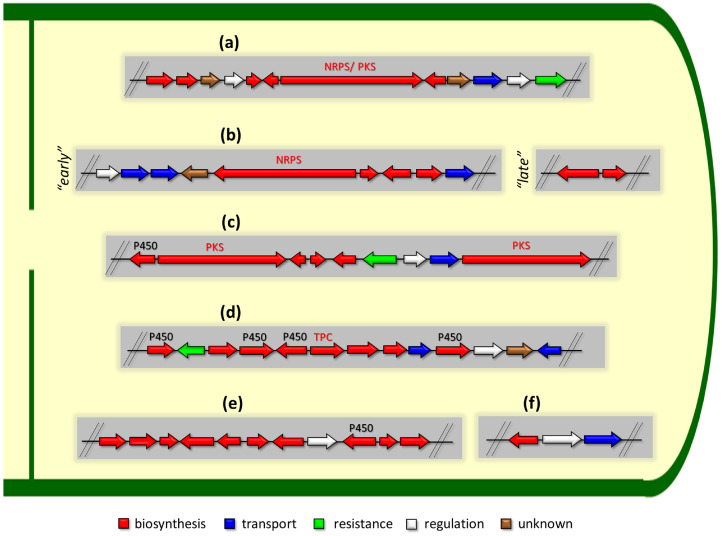
Some examples of the organization of biosynthetic gene clusters (BGCs) for the production of secondary metabolites (SMs) in fungi. (**a**) BGCs for production of SMs based on so-called “central” gene, which encodes one type of megasynthase or another: (i) NRPS (nonribosomal peptide synthetase), (ii) PKS (polyketide synthase), or (iii) NRPS-PKS hybrid. (**b**) “Early” and “late” BGCs for production of cephalosporin C in *Acremonium chrysogenum* [168]. (**c**) BGCs for production of lovastatin in *Aspergillus terreus*: P450—cytochrome P450 [169]. (**d**) BGCs for production of terpenoid SM: TPC—terpene cyclase. (**e**) BGCs for production of meroterpenoid with unique structure. BGC for production of biscognienyne B is given as an example [125]. (**f**) BGC for production of kojic acid in *Aspergillus oryzae* [133]. Gene loci for enzymes of the biosynthetic pathways of the SMs are colored in red; gene loci for protein transporters of biosynthetic products are colored in blue; gene locus for protecting the microorganism from the produced secondary metabolite is colored in green; gene locus for the specific regulator of this biosynthetic pathway is colored in white; locus for gene with unknown function is colored in brown. Genes for backbone enzymes (NRPS, PKS, and TPC) responsible for the production of the core structure of SMs are colored in red.

There are several main types of BGC organization responsible for the biosynthesis of the corresponding types of SMs in fungi (Figure 1 and Figure 3). In most cases, BGCs contain: (i) one or more genes for backbone, or core, enzymes (synthase or synthetase) responsible for the production of the core structure of SMs, and (ii) a number of genes that encode tailoring enzymes for modifying the core compound to obtain a variety of products [1]. The type of core enzyme (or combination thereof) determines the type of secondary metabolite. The BGC can also assemble genes encoding: (iii) transporters, (iv) proteins that mitigate toxic properties, (v) pathway-specific transcription factors, and (vi) genes with as-yet unknown function (Figure 3) [167].

### 4.1. BGCs with Backbone (or Core) Genes for Megasynthases NRPS or PKS

To create two among the four main types of secondary metabolites, fungi use megasynthases, large modular enzymes such as NRPS, nonribosomal peptide synthetase [170], or PKS (polyketide synthase) [171] (Figure 1a,b and Figure 3a). In these modular enzymes, catalytic domains with a number of functions, required for the polymerization of (i) amino acids, including non-proteinogenic acids (in the case of NRPS), or (ii) acyl groups, from acetyl-CoA to malonyl-CoA (in the case of PKS), are assembled into one huge polypeptide chain [172,173]. As a result, individual megasynthases are responsible for 10–50 or more catalytic activities [15]. In a number of bacteria (~10% of cases), polymerization units do not have a modular organization, and catalytic domains are mainly encoded by individual proteins [174]. It is thought that such non-modular polymerization systems for the production of SMs in bacteria served as a prototype for the development of the modular megasynthases NRPS and PKS [174].

Each module of NRPS is a functional building block responsible for incorporating and modifying a single amino acid unit, which can be either canonical proteinogenic (i.e., used in ribosomal synthesis) or non-canonical non-proteinogenic (i.e., never used in ribosomal synthesis) [175,176]. A typical NRPS module consists of: (i) the adenylation (A) domain, for amino acid recognition and activation; (ii) the peptidyl carrier protein (PCP) domain, for transferring an activated amino acid from the A-domain to its cofactor, 4′-phosphopantetheine; and (iii) the condensation (C) domain, to catalyze peptide bond formation [177]. Along with this, the module may contain a set of optional domains with catalytic functions of methyltransferase (MT), β-ketoacyl reductase (KR), epimerase (E), etc. [178]. Specificity of the recognition of one amino acid or another is achieved due to the substrate-binding center of the adenylation domain of the corresponding module [179,180]. In this regard, the term “nonribosomal” code was introduced, referring to the correspondence of 10 amino acid residues in the substrate binding site of the adenylation domain of a particular NRPS module with a specific proteinogenic or non-proteinogenic amino acid [181,182]. More than 500 non-proteinogenic amino acids have now been found in fungi, many of which are used for non-ribosomal peptide synthesis [183,184]. In addition, for the biosynthesis of a number of non-proteinogenic amino acids themselves, an additional BGC is required [185]. Adding such a significant number of “building block” types to the canonical 20 proteinogenic amino acids (the number of which is strictly limited by genetic coding and the rigidly fixed roles of tRNA and aminoacyl-tRNA synthetizes) makes it possible to drastically expand the range of created low-molecular-weight structures [183]. Fundamentally new structures emerging as a result of the use of new building materials on the NRPS platform provide an advantage to the organisms that produce them, and can also be applied to obtain medically significant natural products [186,187,188].

PKS can have, as in the case of NRPS, a complex multi-module structure (type I noniterative PKS) where a single module from a huge enzyme with multiple modules is used to attach the next building block [189,190]. Such enzymes function as a modular linear conveyor line, in which each active site is used only once [191]. However, in fungi, the most common PKS is the iterative type (type I iterative PKS and type II PKS), which, instead of one large megaenzyme, consists of only one module that reuses necessary catalytic domains in a cyclic fashion [192]. After attaching a building block, the polymerization product is transferred to the beginning of the module to attach the next building block, and so on [193]. Such enzymes function as an iterative assembly line in which each active site of the core domains is used as many times as needed to attach the building blocks [194].

Typically, a single PKS module contains three core (minimal) domains: (i) the acyl transferase (AT) domain selects the building blocks to add to the product and transfers them to (ii) the acyl transfer protein (ACP) domain, which loads them for the polymerization product, and (iii) the ketoacyl synthase (KS) domain, which is required for the decarboxylation condensation of the extendable unit (usually malonyl-CoA or methylmalonyl-CoA) with the acyl thioether [195]. There is also iterative AT-less and ACP-less type III PKS in fungi, which is a homodimer with a molecular weight of about 40 kDa and combines all the activities from the essential type I and II PKS domains [196]. Along with minimal domains, the module may contain a set of optional (or tailoring) domains with catalytic functions of thioesterase (TE), methyltransferase (MT), dehydratase (DH), enoyl reductase (ER), β-ketoacyl reductase (KR), etc. [195]. Depending on the presence and number of reducing domains in PKS, they are subdivided into: (i) NR-PKS—non-reducing PKS, the products of which are true polyketides; (ii) PR-PKS—partially contracting PKS; and (iii) FR-PKS—fully reducing PKS, the products of which are fatty acid derivatives. As a result of this diversity of intramodular organization, PKS, along with NRPS, produce an enormously diverse array of natural products in fungi [197].

There are also known cases when more than one corresponding megasynthase is used for the production of NRPS-driven (Figure 1a) or PKS-driven (Figure 1b) secondary metabolites by fungi. For example, two PKSs are used during lovastatin biosynthesis, one of which, LovB nonaketide synthase (EC:2.3.1.161), uses nine building blocks based on acetyl-CoA or manoyl-CoA, and the other, LovF diketide synthase (2-methylbutanoate polyketide synthase; EC: 2.3.1.244), uses two such building blocks [198]. Accordingly, the lovastatin BGC encodes two PKS genes (Figure 3c). There are also numerous examples of BGCs in fungi encoding both NRPS and PKS. This is discussed in more detail in Section 4.3.

### 4.2. BGCs with Backbone (or Core) Gene for Terpene Cyclase

Terpene cyclase (TPC) is used as the core enzyme for the biosynthesis of the third among the four major types of fungal secondary metabolites, terpenoids (Figure 1c and Figure 3d) [199]. In most cases, TPC clusters in the same BGC as its downstream modification enzymes (Figure 3d) [200].

TPCs form the hydrocarbon backbones of terpenoids, which are then modified by tailoring enzymes to produce final natural products [201]. Depending on the initial generation of the carbocation, class I TPK and class II TPK are distinguished [202]. TPC is a catalytic complex that produces cyclic terpenoids from their linear precursors [203]. Terpenoid cyclization reactions are one of the most complex reactions found in nature [204]. Due to the functional diversity of terpene cyclases, various types of cyclic terpenoids are formed from linear precursors, which, in turn, undergo various modifications. Currently, over 80,000 terpenoids are known, which represent about a third of the described natural products [205]. In most cases, the gene for TPC clusters in the same BGC as the genes for its downstream modification enzymes [200]. However, there are a number of examples, such as lanosterol-derived triterpenes/steroids, where the TPC gene is outside the gene cluster for its downstream modification enzymes [206].

### 4.3. Hybrid BGCs with Genes for Different Backbone Enzymes

In addition to biosynthetic clusters encoding only one type of core enzyme, which leads, respectively, to the production of secondary metabolites of the NRPS type, PKS type, or TPC type (Figure 1b–d), there are mixed-type BGCs that contain genes for different types of core enzymes [160]. There are also BGCs with hybrid core genes, for example, for the production of NRPS/PKS hybrids, part of the gene may encode NRPS modules and the other part PKS modules [175]. In such cases, specific interpolypeptide linkers exist at both the C- and N-termini of the NRPS and PKS proteins, which play a critical role in facilitating the transfer of the growing peptide or polyketide intermediate between NRPS and PKS modules in hybrid NRPS-PKS systems [160].

Among the four basic types of SMs in fungi (NRPS, PKS, terpenes, and alkaloids), there are numerous chimeric variants. As a result, the production of such mixed (or hybrid) fungal BGCs results in chimeric secondary metabolites such as NRPS/PKS, NRPS/terpenoid, PKS/terpenoid, or alkaloid/terpenoid hybrids (Figure 1e–h) [67,160,207]. Some (but not all) alkaloids also use core enzymes for their construction [208,209]; for example, ergot alkaloids use NRPS [210,211]. In rare cases, secondary metabolites in fungi result from crosstalk between two separate BGCs [212]. Such an interaction not only increases the structural diversity but also significantly expands the activity spectrum of the produced cross-cluster compounds [212]. NRPS-PKS hybrids (Figure 1e) are among the most common in nature [102]. Such compounds benefit from the combinatorics of products resulting from NRPS and PKS synthesis [175]. It has been shown that more than a third of the clusters encoding megasynthases carry NRPS-PKS hybrids [174].

### 4.4. BGCs without Genes for Canonical Backbone Enzymes (“Wild BGCs”)

In addition to the main types of SMs, in the production of which relatively easily identifiable genes of core and tailoring enzymes are involved (Figure 1a–h), fungi also produce highly active low-molecular-weight compounds that do not have characteristic elements for their “barcoding” (Figure 1i) [213]. BGCs for the production of such SMs do not contain genes encoding canonical “backbone” synthases/synthetases (e.g., NRPS, PKS, TPC); for example, clusters for the production of clavine alkaloids [214], isocyanides [215], NRPS-independent siderophores (NIS) [127], and other [133].

BGCs for the production of clavine alkaloids do not contain NRPS [214], unlike ergot alkaloids, with four genes encoding NRPS [216]. Isocyanides (also called isonitriles) have notable bioactivities that mediate pathogenesis, microbial competition, and metal homeostasis through metal-associated chemistry [215]. For isocyanide production, fungi use non-canonical BGCs (containing the non-canonical core enzyme isocyanide synthase, ICS), which are not detected by standard genome-mining algorithms [217]. However, a targeted bioinformatics study of 3300 fungal genomes allowed 3800 ICS BGCs to be characterized [213]. Hydroxamic siderophores also use NRPS, but recently, an NRPS-independent siderophore (NIS) synthetase pathway has been established for the production of NRPS-independent siderophores [116]. Five functional types of NIS enzymes are classified; all such clusters also lack the core canonical gene [57]. The BGC for kojic acid production does not contain genes encoding both core enzymes and characteristic tailoring enzymes (Figure 3f) [133]. The lack of conserved signature sequences makes such BGCs almost impossible to detect as a result of genomic mining using current bioinformatic approaches [218]. The only way to detect such clusters is through an experimental approach. For example, the BGC of kojic acid in *Aspergillus oryzae* was identified as a result of a reverse genetic method combined with a DNA microarray technique [133].

Currently, most of our knowledge about BGCs is formed in silico [219]. As a result of the application of bioinformatics technologies, tens of thousands of BGCs have been found in fungal genomes, for most of which the products are still unknown [160]. Along with this, for all secondary metabolites from bacteria, fungi, and plants, fewer than two thousand corresponding BGCs have been experimentally characterized [220,221]. As a result, our knowledge of “wild” clusters (without characteristic core and tailoring enzymes) is much narrower than that of BGCs containing these elements.

There are also “canonical” BGCs without genes for core enzymes. This is due to the fact that genes encoding canonical core enzymes for such clusters are localized outside the cluster, in the other part of the genome. For example, the “late” beta-lactam BGC contains only genes for tailoring enzymes (CefEF and CefG), while the core enzyme for this biosynthetic pathway clusters in the “early” beta-lactam BGC, which is located on a different chromosome (Figure 3b) [222,223].

### 4.5. Tailoring Enzymes (Enzymes for Modifying the Core Structure)

The final products of biosynthetic secondary metabolism pathways are often significantly modified as a result of enzymatic activities such as heterocyclization, epimerization, oxidative hydroxylation, methylation, oxidative crosslinking, the addition of sugars, translocation, and other modifications [224]. Some tailoring enzymes assemble as optional domains within megasynthase modules; other tailoring enzymes act in trans during megasynthase work, recognizing the modules required by protein–protein interactions [224]. For example, the trans-acting polyketide enoyl reductase LovC (lovastatin enoyl reductase; EC: 2.3.1.161) specifically reduces three out of eight polyketide intermediates (triketides, tetraketides, and hexaketides) during nonaketide synthase LovB activity in lovastatin biosynthesis [198]. As a result of such cis- and trans-activities, the core polymerization product may contain, after the release, a significant number of modifications. The release of the core scaffold process itself is quite complex; it can proceed using various mechanisms [225], the implementation of which may also require special enzymes encoded in the corresponding BGC. For example, in the biosynthesis of lovastatin, thioester hydrolases LovG (dihydromonacolin L-[lovastatin nonaketide synthase] thioesterase; EC: 3.1.2.31) is required to release from nonaketide synthase LovB its final product, dihydromonacolin L [226]. After the backbone, or core, enzymes create a core scaffold (with cis- and possibly trans-modifications), a third group of tailoring enzymes transform its structure, resulting in a variety of end products. Thus, in addition to the genes for core enzymes, BGCs contain genes for various biosynthetic enzymes, trans-acting with core enzymes, helping to release or modify the released core products (Figure 3).

For example, in *A. chrysogenum*, after NRPS, which is called PcbAB or ACV (δ-[L-α-Aminoadipoyl]-L-Cysteinyl-D-Valine) synthetase (EC: 6.3.2.26), polymerizes the LLD-ACV tripeptide δ-(L-α-aminoadipoyl)-L-cysteinyl-D-valine, a series of enzymatic reactions occur, catalyzed by enzymes from beta-lactam BGCs, resulting in the production of cephalosporin C (CPC). First, PcbC (isopenicillin N-synthase (EC: 1.21.3.1)), as a result of a dioxygenase reaction, cyclizes this tripeptide to isopenicillin N (IPN); then, cefD1 (isopenicillin N-CoA synthetase (EC: 5.1.1.17)), and cefD2 (isopenicillin N-CoA epimerase (EC: 5.1.1.17)) catalyze reactions leading to the epimerization of IPN to penicillin N (penN); finally, enzymes of the “late” beta-lactam BGC, CefEF (deacetoxycephalosporin C synthetase (penicillin N expandase, EC: 1.14.20.1)/deacetoxycephalosporin C hydroxylase (EC: 1.14.11.26)), and CefG (deacetylcephalosporin-C acetyltransferase (EC: 2.3. 1.175)), carry out reactions leading to the formation of CPC [168,227,228].

A distinctive feature of BGC in terpenoid biosynthesis is the presence among the genes for tailoring enzymes of a significant number of genes for cytochrome P450 mono-oxygenases (CYP450), NAD(P)+, and flavin-dependent oxidoreductases that generate the final bioactive structures (Figure 3d) [63]. Individual members of the CYP450 superfamily catalyze various stereospecific modifications at various positions in the core structures of terpenoids, as a result of which their biological activity can significantly increase [229,230]. The most important modification catalyzed by CYP450 is oxidative hydroxylation, which makes the compound more hydrophilic [231]. Clustered NAD(P)+ and flavin-dependent oxidoreductases are required for CYP450 to function as partners in the electron transfer chain [232].

In addition to terpenoids, CYP450s are also used to modify other types of fungal secondary metabolites based on NRPS, PKS, and NRPS-PKS activities and meroterpenoids [233]. For example, LovA (CYP68R1, dihydromonacolin L/monacolin L hydroxylase; EC: 1.14.14.124, EC: 1.14.14.125) from the lovastatin biosynthetic pathway sequentially introduces two hydroxyl groups into the backbone (dihydromonacolin L), which leads to: (i) the introduction of the 4a,5-double bond and obtaining monacolin L, which, in turn, (ii) is hydroxylated at C-8 to form monacolin J [234]. The hydroxyl inserted at the C-8 position is then used to incorporate the independently synthesized diketide via a transferase reaction involving LovD (monacolin-J-acid methylbutanoate transferase; EC: 2.3.1.238) to form the final product, lovastatin [235]. However, CYP450s localized separately (without association with any core enzyme of VM biosynthesis) are not always good indicators for the search for biosynthetic clusters of secondary metabolism, since they are used not only to build secondary metabolism, but also for the biosynthesis of structural components and in signaling networks, and are instrumental in xenobiotic detoxification [229,236,237,238]. There are currently about 400 CYP families (namely, CYP51-CYP69, CYP501-CYP699, and CYP5001-CYP6999) [239], which exceeds the diversity in the number of families of representatives of this protein superfamily in bacteria (333 CYP families), plants (127 CYP families), vertebrates (19 CYP families), and insects (67 CYP families) [229]. Due to this variety in the most important enzymatic components of fungi, as well as the lack of data on structural and functional relationships for the vast majority of CYP450, the presence of their genes is only a signal for a possible search for BGCs.

### 4.6. Transporter Genes of BGCs

It also turns out that, together with the genes for the biosynthesis of a secondary metabolite, the genes necessary for the transport of the final product or its intermediates can be clustered [26,168,240,241]. Such transport can occur both for the removal of the end product from the cell, and for the transport of metabolic intermediates between different compartments of the cell, where the stages of biosynthesis take place [168,169,242,243]. For example, in *A. chrysogenum*, the first steps in the biosynthesis of cephalosporin C (CPC), leading to the biosynthesis of IPN, occur in the cytoplasm; then, in the peroxisome, epimerization of IPN to penicillin N (penN) occurs; the final conversion of penN into the target SM, CPC, occurs again in the cytoplasm [244]. For this purpose, in the “early” BGC of beta-lactams, there are special genes for transporter proteins that carry out active transport of the corresponding intermediates: first, as a result of the activity of the CefP transporter, IPN enters peroxisome from the cytoplasm [245]; then reactions occur in the peroxisome, leading to the epimerization of IPN to PenG [227], which then, as a result of the activity of the CefM transporter [246], moves from the peroxisome to the cytoplasm, where it undergoes further transformations.

### 4.7. Gene for Resistance of BGCs

Another important class of genes found in BGCs are resistance genes against the directly synthesized compound (Figure 3). The physiological basis of this strategy is that many high-yielding natural products, such as antibiotics or statins, can harm the host organism by acting on microorganisms with similar biochemistry [1]. This is why it is necessary to “defend” against a number of compounds created by the microorganism itself [169,247]. Currently, three main defense strategies for BGC resistance genes have been classified. They are associated with: (i) placement in the BGC of an additional copy of the gene encoding the target protein, which is inhibited by the produced metabolite; (ii) the active transport of a “hazardous” substance from the cell; and (iii) the coding of an enzyme that detoxifies the final highly active antimicrobial product [142]. For example, the “early” beta-lactam BGC also contains the gene for the CefT transporter, which serves in the active transport of CPC and its intermediates, such as IPN, PenN, deacetoxycephalosporin C (DAOC), and deacetylcephalosporin C (DAC), out of the cell [168,248]. In the BGC for the production of lovastatin (LOV), a compound that affects the ergosterol biosynthesis of competing fungi (and potentially affects endogenous ergosterol biosynthesis), *lovR* is clustered, representing an additional copy of the gene encoding 3-hydroxy-3-methyl glutaryl coenzyme A reductase (EC: 1.1.1.34), which is inactivated by LOV as a result of irreversible binding.

### 4.8. Pathway-Specific and Cross-Cluster Regulators of BGCs

Finally, in addition to genes for biosynthesis, transport, and resistance, there is a fourth class of genes, often, but not always, found in BGCs, that are responsible for pathway-specific regulation of the BGC itself and/or of other BGCs, in the case of cross-regulation [166]. Such genes encode transcription factors that are able to modulate the effect of signals perceived and reproduced by global regulators and occur in more than half of the currently known BGCs [249]. These factors can act as positive regulators during the signal amplification stage [166]. There are also negative pathway-specific regulators leading to downregulation of the BGC; they are more common if genes for two regulators are clustered in the same BGC and one regulator is positive while the other is negative [166]. However, there are regulators that can be positive for some BGC genes and negative for others. For example, in *A. chrysogenum*, the early BGC beta-lactam cluster contains a gene for the CefR regulator, which is both a negative regulator for the *cefT* transporter gene from the early BGC and a positive regulator for the *cefEF* biosynthetic gene from the late BGC [250]. Thus, CefR from the early BGC beta-lactam cluster is a pathway-specific regulator for *cefT*, and a cross-cluster regulator for *cefEF*. Such a differential effect of CefR on the expression of beta-lactam BGCs in *A. chrysogenum* allows, on the one hand, the biosynthesis of CPC to be intensified (as a result of upregulation of one of the key biosynthetic genes), and on the other hand, for a reduction in the “leakage” of intermediates from the cell (such as IPN, PenN, DAOC, and DAC) and their redirection toward producing the target metabolite, CPC.

## 5. Methods for Improving Fungal Strains for the Production of Secondary Metabolites

**Figure 4:**
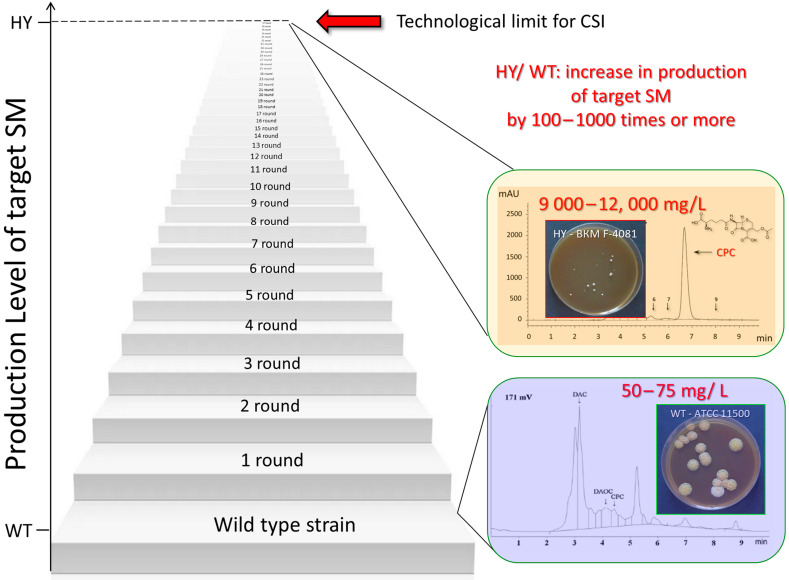
Classical strain improvement (CSI) program for increasing the production of a target secondary metabolite (SM) in filamentous fungi. The wild-type (WT) strain in the first round is subjected to random mutagenesis at a sublethal level. The clones obtained as a result of such exposure are screened according to the level of production of the target SM. Typically, most clones will show less or equal activity compared to the initial strain; however, clones with higher activity than the original strain are also detected. The clone with the highest activity is used for new random mutagenic exposure (second round) followed by screening and selection of the most active strain. This procedure is repeated, as a rule, several tens of times, until the next mutagenic effect makes it possible to obtain more active clones. This stage corresponds to the technological limit of the method. The high-yielding (HY) strain obtained at the final (or one of the last) stage of mutagenesis is used for industrial production of the target SM. As an example, production is shown in the wild-type strain *A. chrysogenum* WT (ATCC 11550, CPC production—50–75 mL/L, [256]) and in the strain *A. chrysogenum* HY (RNCM F-4081D, CPC production—9000–12,000 mL/L, [257]) derived from *A. chrysogenum* WT as a result of the CSI program.

Natural fungal isolates, the so-called wild-type (WT) strains, produce a limited amount of the target SM, which is insufficient for industrial production. In this regard, over the past 70–80 years, improved high-yield (HY) producers have been created, in which the yield of the target SM is increased by 100–1000 or more times. There are two principal approaches to improve the production of SMs in fungal strains: (i) an approach using genetic and metabolic engineering methods to introduce targeted changes in the resulting recombinant strains [50,251,252], and (ii) so-called classical strain improvement (CSI) based on random mutagenesis and subsequent screening of the resulting mutants with improved production of target SMs [253]. Currently, all industrial fungal producers of SMs have been obtained using CSI programs, or as a result of modifications introduced into CSI strains [253] (Figure 4). The essence of CSI programs is that a mutagenic effect is applied to the natural producer of a promising SM. Various agents are used for this, such as chemical mutagens, UV, and irradiation. A sublethal dose of mutagenic effects is selected; the obtained clones are screened according to the level of production of the target SM. Typically, most clones show similar or lower activity; however, there are clones that have higher activity than the WT strain. The most active clone is used for a new mutagenic effect (second round of mutagenesis); the obtained clones are screened, and among them, the most active one is selected. This process is repeated several dozen times, which leads to a multiple-fold increase in the production of the target SM. Along with the increase in production, concomitant mutations accumulate. Therefore, strains improved in this way usually have lower viability than WT strains. This can be expressed as a slowdown in the growth rate, a decrease in the size of the colonies on agar media, a decrease in biomass during submerged cultivation, an increase in stress resistance, and many other complications [223,254,255]. As a result, after several tens of rounds of mutagenesis, a stage begins whereby, after the next mutagenic effect, it is no longer possible to obtain more active clones. This stage corresponds to the technological limit of the method. For industrial production, a strain obtained at the last or one of the penultimate stages of mutagenesis is used (if the strain obtained at the last stage is not viable enough for biotechnological application).

The most important (but not the only) event that occurs at the molecular level in CSI is the upregulation of biosynthetic genes, tens and hundreds of times [258,259]. Significant changes also occur at the level of primary metabolism; for example, they can occur at the level of the biosynthesis of precursor amino acids, in the case of NRPS [48]. Other important events in the creation of HY strains may be associated with changes in the physiological and morphological state of the cell, and its life cycle, which are necessary for high-yield fermentation.

Numerous works on changing the production of SMs in fungi as a result of only direct genetic engineering manipulation of wild-type strains have not yet led to the creation of industrial producers. For example, an attempt to achieve the production of penicillin G (PenG) as a result of the heterologous expression of its BGC in *Saccharomyces cerevisiae* was only of theoretical significance, since after the optimization of all conditions, the maximum yield of PenG was 70–280 μg/L [260], while industrial strains improved via classical methods produce more than 50 g/L of PenG [59]. Such a powerful method as genetic engineering does not make it possible to create industrial fungal producers of SMs from natural isolates, since the transformation of a WT strain into HY requires not one or two, but a whole range of changes, including at the level of the global regulation of secondary metabolism (for more details, see Section 7.3).

In this regard, a promising approach is the combination of classical and genetic engineering methods. For example, HY strains from CSI programs (such as, *P. chrysogenum* DS17690) after inactivation of their most active host BGCs can be used as recipient strains for the heterologous expression of target BGCs. So, in a recent work, *P. chrysogenum* was modified for industrial use, in which the four highly expressed biosynthetic gene clusters required to produce penicillin, roquefortine, chrysogine, and fungisporin were removed [58]. One of the few examples of a successful combination of metabolic engineering and CSI is the industrial production of pravastatin, a cholesterol-lowering drug belonging to the statin class [261]. In industry, the drug pravastatin has traditionally been produced via a semi-synthetic method. For this, compactin (Table 1) is produced in improved *Penicillium citrinum* strains [262], and then, converted to compactin in a one-step reaction using biocatalysts [263]. For in vivo pravastatin production, compactin BGC from *P. citrinum* and cytochrome P450 from *Amycolatopsis orientalis* (CYP105AS1, used to catalyze the final compactin hydroxylation step), fused to a redox partner, were transferred into β-lactam-negative *P. chrysogenum* DS50662 [261]. Numerous additional manipulations, such as the deletion of esterase activity, yielded more than 6 g/L pravastatin on a pilot production scale.

## 6. Hierarchical Organization of the Secondary Metabolism Regulation System in Fungi

**Figure 5:**
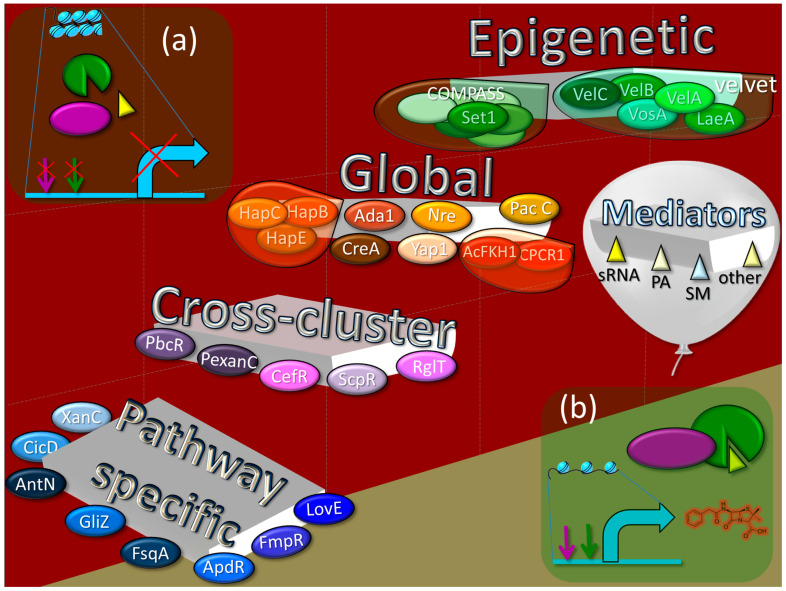
Levels of regulation of the biosynthesis of secondary metabolites (SMs) in fungi. (**a**) A particular gene from a biosynthetic gene cluster (BGC) is “silent” under normal physiological conditions, in the absence of a stimulating effect from the regulatory system. The absence of their transcription is associated both with the absence of the necessary transcription factors and with the functional state of these loci, which are in the form of heterochromatin. (**b**) The activation of a gene from the BGC as a result of coordinated regulation at the levels of (i) pathway-specific, (ii) cross-cluster, (iii) global cell, and (iv) global secondary metabolism (at the epigenetic level), (v) mediated by regulatory molecules. The antibiotic penicillin G is given as an example of a SM synthesized due to BGC activation. Green and purple arrows show transcription factor binding sites, curved cyan arrow indicates the start of transcription. Cross symbols on these arrows indicate that binding sites are not available for transcription factors. Pathway-specific regulators: ApdR—Zn(II)_2_Cys_6_ regulator for aspyridone A and B BGC (PKS/NRPS hybrid); FsqA—Zn(II)_2_Cys_6_ regulator for fumisoquin BGC (NRPS); FmpR—Zn(II)_2_Cys_6_ regulator for fumipyrrole BGC (NRPS); CicD—regulator with Myb-like DNA-binding domain for cichorine BGC (PKS); AntN—Zn(II)_2_Cys_6_ regulator for aspercryptin BGC (NRPS); XanC—bZIP transcription factor for xanthocillin BGC (isocyanide synthase); GliZ—Zn(II)_2_Cys_6_ regulator for gliotoxin BGC (NRPS); LaeA—Zn(II)_2_Cys_6_ regulator for lovastatin BGC (PKS). Cross-cluster regulators: PbcR—Zn(II)_2_Cys_6_ regulator for ent-pimara-8(14),15-diene BGC (terpenoid), which also downregulates penicillin cluster, two putative PKS clusters, and one putative NRPS cluster and upregulates one siderophore BGC; PexanC—bZIP transcription factor for upregulation of xanthocillin BGC, which also upregulates citrinin BGC; ScpR—transcription factor with C_2_H_2_-type zinc finger for upregulation of fellutamide B BGC (NRPS), which also upregulates asperfuranone BGC (PKS); RglT—Zn(II)_2_Cys_6_ transcription factor, whose gene is localized outside the gliotoxin BGC (NRPS), for which it is a positive regulator; CefR transcription factor with nuclear targeting signal that downregulates some genes from “early” beta-lactams BGC (NRPS) and upregulates some genes from “late” beta-lactams BGC. Global regulators: CreA (Cre1)—C_2_H_2_-type zinc finger transcription factor for glucose catabolite regulation; PacC—C_2_H_2-_type transcription factor with three zinc fingers for pH regulation; Nre (or AreA)—GATA transcription factor with single Cys_4_ zinc finger for nitrogen regulation; Ada1—C_2_H_2_-type transcription factor for control of asexual development; Yap1—bZIP-containing transcription factor for antioxidant response; HapB, HapC, and HapE—transcription factors from CCAAT-binding complex for regulation of redox status and iron starvation; CPCR1—RFX transcription factor for morphological development; AcFKH1—forkhead transcription factor for regulation of morphogenesis. Epigenetic regulators (including global regulators of secondary metabolism). Velvet complex: LaeA—S-adenosylmethionine-dependent histone methylase for chromatin remodeling; VelA (VeA), VelB, VelC, and VosA—components of so-called velvet complex with velvet domain for interacting with each other and with LaeA in the fungal nucleus. COMPASS complex—complex associated with Set1: Set1—histone-lysine N-methyltransferase (H3 lysine-4-specific). Mediators: sRNA—small non-coding RNA; PA—polyamine; SM—secondary metabolite.

The effective production of the target SM requires the implementation of a whole complex of events at the molecular level that occur in the fungal cell after the receipt of a particular signal. Ultimately, whether or not the synthesis of one secondary metabolite or another will occur depends on the complex and hierarchical system of regulation that functions in the fungal cell (Figure 5).

At the lowest level in this hierarchy are the pathway-specific regulators, transcription factors whose genes are localized within the BGCs they regulate. Thus, if the BGC is “silent”, its pathway-specific regulator is also not working. Slightly higher in this hierarchy are cross-cluster regulators, which are transcription factors whose genes are clustered in different BGCs than those they regulate. Thus, the cross-cluster regulator can theoretically regulate the “silent” BGC if the cross-cluster regulator’s own BGC is expressed. However, in most cases, cross-cluster regulation does not function without “permission” from the regulatory systems at a higher level. A cross-cluster can simultaneously be a pathway-specific regulator if it regulates not only foreign but also its own BGC. For a higher level of regulation, global cell regulators are branched. They are transcription factors that have binding sites for promoters of numerous genes and coordinate, in response to signals (such as light, temperature, pH, carbon, nitrogen, iron, etc.), various cell processes, including the biosynthesis of SMs. The global regulator genes are not associated with BGCs, so their functioning does not depend on the expression of any BGCs. An even higher level of regulation is associated with the epigenetic status of BGCs [54]. In the fungal cell, there is a special system of global regulation of secondary metabolism, which, in response to internal or external signals, remodels chromatin in BGC-containing loci, which are mosaically scattered over chromosomes. In the absence of appropriate signals (Figure 2a and Figure 5a) the majority of BGCs are in a heterochromatic state, which prevents the production of the corresponding SMs. Upon receipt of the appropriate signal, the remodeling system converts the required BGC-containing loci from heterochromatin to euchromatin, which makes binding sites available for global, cross-cluster, and pathway-specific regulators (Figure 5b). In addition to the described main levels of regulation, represented by transcription factors and the chromatin remodeling system in the fungal cell, there are a number of low-molecular-weight compounds that can indirectly affect the production of secondary metabolites, enhancing or weakening regulation.

### 6.1. Pathway-Specific Regulation

Pathway-specific regulatory proteins are transcription factors whose genes are localized within the BGC they regulate [264]. The basic function of such transcription factors in a single BGC is as specific positive regulation. As a rule, the genes for these proteins are not expressed under conditions whereby their BGC is not induced (“silent”) [166]. They are at the lowest level in the hierarchical structure of regulation (Figure 5), and they start working when the cluster “wakes up” under the influence of regulators located at higher levels [166]. Pathway-specific regulators usually control the expression of all the genes of their BGCs, including their own, which can lead to a signal amplification cascade [164]. However, there are examples whereby not all BGC genes in the promoter regions have sites for binding their pathway-specific regulators [169]. Some examples of pathway-specific regulators include: (i) ApdR—Zn(II)_2_Cys_6_ regulator for aspyridone A and B (PKS/NRPS hybrid) from *A. nidulans* [265]; (ii) FsqA—Zn(II)_2_Cys_6_ regulator for fumisoquin (NRPS) from *A. fumigatus* [266]; (iii) FmpR—Zn(II)_2_Cys_6_ regulator for fumipyrrole from *A. fumigatus* (NRPS) [267]; (iv) CicD—regulator with Myb-like DNA-binding domain for cichorine from *A. nidulans* (PKS) from *A. nidulans* [268]; (v) AntN—Zn(II)_2_Cys_6_ regulator for aspercryptin from *A. nidulans* (NRPS) [269,270]; (vi) XanC—bZIP transcription factor for xanthocillin (isocyanide synthase) from *A. fumigatus* [271]; (vii) GliZ—Zn(II)_2_Cys_6_ transcription factor from *A. fumigatus* for positive regulation of gliotoxin BGC (NRPS) [272]; and (viii) LaeA—Zn(II)_2_Cys_6_ transcription factor from *A. terrius* for lovastatin BGC (PKS) [259].

### 6.2. Cross-Cluster Regulation

The regulator from *Aspergillus nidulans* PbcR, encoded by the *pbcR* gene, upregulates the transcription of BGC (where this gene is localized) for the production of a diterpene, ent-pimara-8(14),15-diene [273]. However, it also upregulates the siderophore transporter genes *mirA* and *mirB* and downregulates four other BGCs (penicillin cluster, two putative PKS clusters, and one putative NRPS cluster). Thus, in the case of the production of ent-pimara-8(14),15-diene in *A. nidulans*, there is a decrease in the consumption of primary resources for alternative secondary metabolism, and the system of iron delivery to cells is intensified [166,273]. PexanC, a bZIP transcription factor from the xanthocillin BGC of *Penicillium expansum*, not only upregulates the xanthocillin BGC, but also activates the expression of *ctnA*, the pathway-specific regulator of the citrinin BGC, and increases the production of citrinin [27]. ScpR is a transcription factor with a C_2_H_2_-type zinc finger for the upregulation of fellutamide B (NRPS) in *A. nidulans* [274], and also activates the silent asperfuranone cluster with PKS by upregulating its pathway-specific regulator, AfoA [275]. RglT is a Zn(II)_2_Cys_6_ transcription factor from *A. fumigatus*, and its gene is localized outside the gliotoxin BGC (NRPS), for which it is a positive regulator [276]. The gene for the transcription factor CefR from *Acremonium chrysogenum* is localized within the so-called “early” beta-lactams BGC (NRPS). CefR downregulates some genes from the “early” beta-lactams BGC (for example, the *cefT* transporter gene) and upregulates the gene for biosynthesis, *cefEF*, from the “late” beta-lactams BGC, which is localized on a different chromosome [250].

### 6.3. Global Regulation

Cre1 (CreA) is a C_2_H_2_-type zinc finger transcription factor for glucose catabolite regulation, e.g., in *Acremonium chrysogenum* [277,278]; PacC is a C_2_H_2_-type transcription factor with three zinc fingers for cellular pH homeostasis [228], e.g., in *A. nidulans* [279]; Nre (or AreA) is a GATA transcription factor with single Cys_4_ zinc finger for nitrogen regulation [280,281], e.g., in *A. nidulans* [281,282]; Ada1 is a C_2_H_2_ type transcription factor for the control of asexual development, e.g., in *Fusarium verticillioides* [283]; Yap1 is a bZIP-containing transcription factor for redox status regulation and antioxidant response [284], e.g., in *Fusarium graminearum* [285]; HapB, HapC, and HapE are transcription factors for the regulation of redox status and iron starvation from the CCAAT-binding factor (AnCF in *Aspergillus nidulans*, [286]); CPCR1 is an RFX transcriptional factor nonconventional modes of DNA recognition for the morphological development of fungus cells, such as hyphal fragmentation for the formation of arthrospores in *A. chrysogenum* [287], or linking cell division with cellular differentiation during morphogenesis, mainly in the process of conidiation and growth under yeast formed in the opportunistic human pathogenic fungus *Penicillium marneffei* (for RfxA, a CPCR1 ortholog) [288]; AcFKH1 is a forkhead transcription factor associated with the RFX transcription factor CPCR1 for morphogenesis (arthrospore formation), e.g., in *A. chrysogenum* [289].

### 6.4. Epigenetic Regulation

A variety of elegant natural models are described that operate at the level of epigenetic protein complexes, remodeling chromatin in response to external or internal signals [290]. One such machine for chromatin remodeling in eukaryotic cells is the ATP-dependent chromatin remodeling complex SWI/SNF [291,292]. This complex is also important for fungi in the response of their primary metabolism to external influences [293]. However, fungi also use special complexes of protein machines to regulate the status of chromatin for BGCs, such as the so-called velvet complex, based on LaeA and VelA global regulators of fungi secondary metabolism [57,164,294]. LaeA is an S-adenosylmethionine-dependent histone methylase for chromatin remodeling, a global regulator of secondary metabolite biosynthesis [169,295]. In *A. fumigatus*, LaeA positively controls the expression of 20% to 40% of major SM biosynthesis genes, such as nonribosomal peptide synthetases, polyketide synthases, and P450 monooxygenases [296]. A whole-genome comparison of the transcriptional profile of wild-type, *ΔlaeA*, and complemented control strains showed that genes in 13 of 22 secondary metabolite BGCs were expressed at significantly lower levels in the *ΔlaeA* mutant [296]. The knockdown of *laeA* also results in a loss of characteristic pigmentation in fungal strains associated with BGCs positively regulated by LaeA [31]. VelA (VeA), VelB, VelC, and VosA are components of the so-called velvet complex with a velvet domain for interacting with each other and with LaeA in the fungal nucleus [297].

Another important player that can change the epigenetic status of BGC-containing loci is the COMPASS (complex associated with Set1) complex [298]. This complex is a conserved eukaryotic transcriptional effector that acts epigenetically through the methylation of lysine 4 of histone 3 (H3K4) and is responsible for multiple functions, such as the regulation of homothallic mating silencing, ribosomal DNA silencing, telomere length, and subtelomeric gene expression in yeast [299,300]. It was shown that the deletion of the Bre2/ASH2 homolog *cclA*, a critical member of the COMPASS complex, in *Aspergillus nidulans* activates the expression of the cryptic BGCs of emodin, monodictyphenone, and their derivatives [298]. The deletion of *cclA* in *Aspergillus fumigatus* decreased growth but increased production of several SMs, including gliotoxin.

### 6.5. Possible Role of Mediators in Fungal Cell Regulation

Mediators are low-molecular-weight compounds that affect the biosynthesis of secondary metabolites in fungal cells. Such compounds include polyamines (PAs). It was shown that the introduction of exogenous PAs such as 1,3-diaminopropane or spermidine during the fermentation of HY strains, obtained via CSI, can further increase the production of the target SMs, by 10–45% [42,44,301]. Moreover, an additional increase in production is even observed for strains that have reached the technological limit of the method in the process of the CSI program [42,44] (we provide more information on this in Section 5). In this regard, the effect found on the increase in production in HY strains of pharmaceutically significant drugs (such as PenG, CPC, and lovastatin) with the addition of relatively cheap PA could have a biotechnological application. It is assumed that PAs affect the system of global regulation of the secondary metabolism of fungi, since their addition is accompanied by the upregulation of *laeA*, which can also lead to the observed upregulation of biosynthetic genes in the BGCs of target SMs [44,301]. It has also been shown that, under certain conditions, polyamines can lead to the downregulation of *laeA*, which leads to downregulation of the target BGC and a decrease in the production of the corresponding secondary metabolite [169].

For prokaryotes, the role of small non-coding RNA (sRNA) in the production of SMs has been shown [302]. It is possible that sRNAs also play a mediating role in fungi. This is indicated by a number of indirect factors; for example, in phytopathogenic fungi, when plants are infected, the amount of sRNA significantly increases in parallel with the biosynthesis of secondary metabolites [145,303]. At the same time, in most works, the role of fungal sRNAs in phytopathogenicity is considered only from the point of view of their interaction with the plant microbiome or with the plant itself, but not from the point of view of their impact on the biosynthesis of its own virulence factors, in particular, secondary metabolites [304]. It is also possible that the mediating effect of sRNA on the regulation of the secondary metabolism of fungi is realized in the framework of the recently discovered phenomenon of “strand commutation” [305].

An important role in regulation can be played by the secondary metabolites themselves, since at least some of them are directly involved in the control of their own production, like the primary metabolites, via a feedback mechanism [35]. Several types of inhibition of SMs’ own biosynthesis are known, which can occur either at the initial, intermediate, or final stages of biosynthesis. Thus, in *Claviceps*, the ergot alkaloids agroclavine and elymoclavine inhibit their own biosynthesis at the first stage [306,307]. Elymoclavine also inhibits a later enzyme, chanoclavine-I-cyclase [308]. In *Penicillium stoloniferum*, mycophenolic acid is, on the contrary, an inhibitor of the final stage of its own biosynthesis [309].

It is possible that there are other low-molecular-weight compounds that have a significant effect on the functioning of the regulatory system of secondary metabolism in fungal cells, which will be discovered as a result of expanding the research arsenal, in particular, with the involvement of modern multiomics approaches.

## 7. Regulation of SM Production in Fungi According to the Piano Principle

In the previous section, we analyzed the currently known main levels at which such a complex and hierarchical process as the regulation of secondary metabolism in fungi functions. In this section, we will compare the various elements of the regulatory system with the equivalent parts of a piano. Such a comparison seems justified because, like a fungal cell that is finely tuned to produce a particular SM when it receives the appropriate signal, a piano is designed and tuned to produce a certain sound when a certain key is pressed. We will also discuss what changes occur at the molecular level in a fungal cell, a unique natural tool, when a cell factory is created from a natural isolate to produce a certain secondary metabolite that a person needs, for example, a drug. How should the fungal cell, this natural piano, be hewn and remade so that it emits only one very loud sound upon pressing the key of the target BGC.

### 7.1. Piano Model for Describing the Principle of SM Production

Like a fungal cell, which has about a hundred BGCs, a typical piano has 88 keys (an organ can have several hundred keys). Also, like a fungal cell, in which, without the absence of signals, BGCs are silent and no synthesis of SMs occurs, the piano makes no sound without the influence of a pianist. Apart from this, like a fungal cell, in which a specific signal leads to the expression of one BGC or another and the synthesis of the corresponding SM, when a certain piano key is pressed, the corresponding sound is emitted.

**Figure 6:**
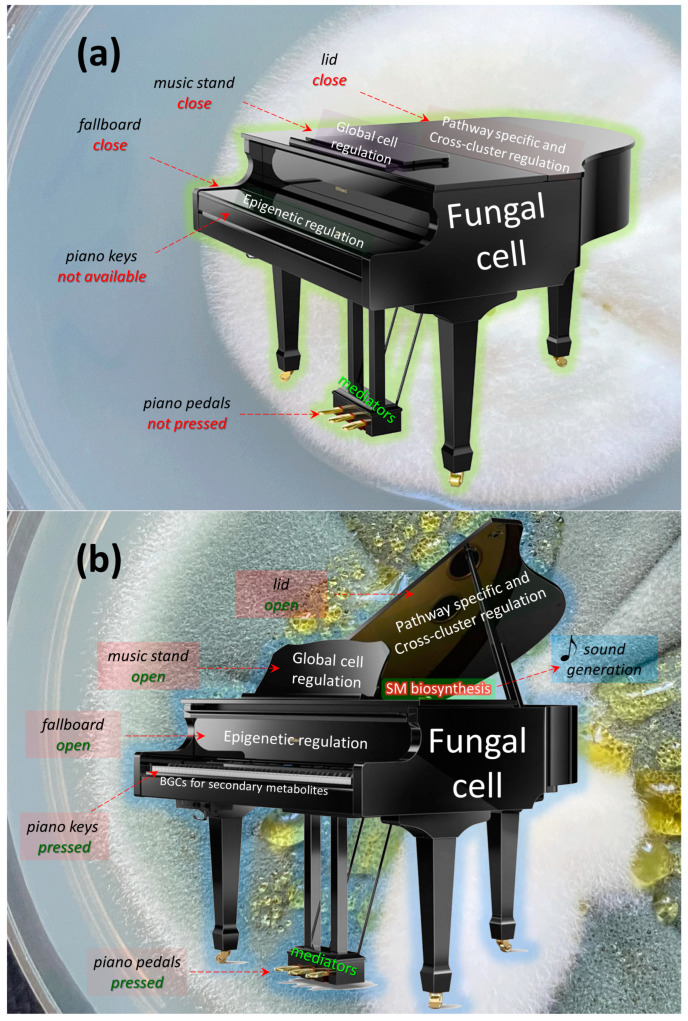
Regulation of the production of secondary metabolites (SMs) in the fungal cell according to the piano principle. (**a**) Under initial physiological conditions, SMs are not synthesized in the fungal cell (the piano does not make sound). The biosynthetic gene clusters, BGCs (piano keys), required for the production of SMs, are located in the heterochromatin regions and are inaccessible for gene expression (the piano keys are not available for pressing), since there is no activation by epigenetic global regulators of SMs capable of transferring the BGC loci to the euchromatin state (the fallboard is closed). There is also no activation from the side of the global regulators of the cell (the music stand is closed), as well as cross-cluster and path-specific controls (the piano lid is closed). Since there is no sound (SM biosynthesis), this process is not affected by mediators (pressing the piano pedals). The background of the figure is filled with a photograph of a colony of *Penicillium chrysogenum* STG-117 (MW556011.1) after cultivation on Czapek Dox agar (CDA) medium for 5 days at 26 °C. The microorganism is at the trophophase stage, since the colony is unstained and the synthesis of secondary pigment metabolites has not yet occurred. (**b**) For SM biosynthesis by a fungal cell, after receiving an appropriate signal (for example, a pianist with sheet music has arrived), the epigenetic regulatory system transfers the corresponding BGC loci from the heterochromatic state to the euchromatic state (the piano lid opens). This opens the possibility for gene expression of the corresponding cluster (the ability to press the piano keys). However, gene expression is also controlled by global cell regulators (the pianist opens the music stand and sheet music is placed on it, which determines the order in which the keys are pressed), as well as cross-cluster and path-specific controls (opening the piano lid). All of this leads to pressing a specific key (BGC), which leads to the appearance of the sound corresponding to it (synthesis of the target secondary metabolite). The sound of a single key can be changed, for example, by pressing the forte or piano pedal (also, the complex effect of mediators can increase or, conversely, reduce the production of the target SM). The background of the figure is filled with a photograph of a colony of *P. chrysogenum* STG-117 after cultivation on CDA medium for 12 days at 26 °C. The appearance of a characteristic pigment associated with the biosynthesis of chrysogine and sorbicillin SMs indicates the transition of the microorganism to the idiophase, which is coproduced through the synthesis of SMs.

If we continue this analogy at the level of regulation, in the absence of signals, (i) BGCs for the production of SMs are in a state of heterochromatin (the piano fallboard is closed); additionally, at these loci, (ii) the binding sites for global regulators are not available (the music stand is closed), (iii) the binding sites for cross-cluster and pathway-specific regulators are also not available (the piano lid is closed), (iv) and there is no influence of mediators (the piano pedals are not pressed) (Figure 6a). As a result, there is no production of SMs (it is impossible to press a key and no sound is emitted).

In order to trigger the production of a secondary metabolite (extract a sound) after receiving a signal, at the first stage, it is necessary to influence the epigenetic regulatory system and transfer the BGC to the appropriate locus from heterochromatin to euchromatin status (open the keyboard cover) (Figure 6b). This allows, at the next stage, for the implementation of the program of global regulators (opening the music stand and setting the sheet music), to achieve the effect of cross-cluster and path-specific regulation (the change in signal strength from opening/closing the piano lid). As a result of influencing a specific BGC (pressing a specific key), a specific biosynthesis process occurs (a certain hammer strikes its string), leading to the appearance of a corresponding SM (a specific sound is emitted). The level of biosynthesis may differ depending on the effect of a number of low-molecular-weight compounds and mediators (and the characteristics of the sound can be changed by pressing the pedals).

In the current model, a key of the piano is a BGC. The activation of one particular BGC (pressing a key) results in the production of one particular secondary metabolite (sound extraction). A 1:1 ratio is observed both when playing the piano (Equation (1)) and during SM biosynthesis (Equation (2)).

NP_K_:NEs = 1:1 (1)

NA_BGC_:NP_SM_ = 1:1 (2)

where NP_K_—number of pressed keys, NEs—number of emitted sounds, NA_BGC_—number of activated BGCs, and NP_SM_—number of produced SMs (only the final products of the metabolic pathways are taken into account, not the intermediates).

This ratio works in principle for any number of BGCs (keys). For example, pressing three keys results in three sounds (Equation (1)), and the activation of three BGCs leads to the appearance of three different SMs (if only the final products are taken into account, ignoring the intermediates). Just as a pianist can play multiple keys at the same time (for example, playing a chord), multiple BGCs can be activated at the same time and multiple SMs can be produced.

But the following question arises: what in nature presses this key? A pianist presses one finger per key to extract one sound per unit of time. Two fingers extract two sounds, three fingers—three keys—extract three sounds, etc., up to ten fingers—ten sounds. In nature, the a BGC key is pressed by one or several signals that determine the totality of the external and/or internal states of the organism and affect the hierarchical system of regulation.

According to the piano model, the level of the signal’s effect on SM yield can be compared with the way the key is pressed. The very method of pressing the key allows for variation in the characteristics of the sound, such as its strength, duration of sound, attenuation characteristics, and a number of others (Equation (3)).

IP:SP = a(3)

where IP—Impact Level (pressing force, key touch speed, and others), SP—sound parameters (sound intensity, duration, attenuation characteristics, and others), and a—variable parameter.

When a good pianist plays his piano, the relationship between IP and SP, that is, parameter “a” in Equation (3), is under his control. For example, it is possible to quantify both the force of pressing a particular piano key and the strength of the resulting sound. Pressing harder will make the sound stronger; pressing even harder will make it sound even stronger. Finally, there is a pressure level that will result in the loudest possible sound that a piano can extract. This relationship is described in Equation (3). The different SPs when playing a piano are given both by note duration and so-called strokes. Strokes determine the character, timbre, and attack of music, creating musical images. For example, a staccato stroke means that each note must be played clearly, abruptly, and sharply. The finger strikes a note and immediately releases it. Staccato means using 50% of the duration of the note for the sound and 50% for the rest to make up for the unused time of the duration. A legato stroke means that one note should smoothly flow into another. There are also the following strokes: non-legato (the sound must stop before making the next sound), accented non-legato (a louder sound), wedge-shaped staccato (an even shorter staccato duration), tenuto (exceeding the duration of a note), fermato (an irregular increase in the duration notes), French league (starts from a note and goes nowhere; you need to hear the natural fading of the sound), and some others.

Unlike a pianist who feels the ratio of IP and SP, currently, there are no clear experimental data to quantify the relationship between the level of the signal (signals) and the production of most secondary metabolites (Equation (4)).

SP:Y_SM_ = x (4)

where SP—signal power, Y_SM_—yield of secondary metabolite, and x—unknown variable parameter.

This ratio, designated as unknown “x” in Equation (4), can change significantly with changes occurring in the microorganism at the molecular level, especially at the level of regulation. For example, the value of “x” decreases in the process of improving strains, as the Y_SM_ (divisor) increases. In particular, the Y_SM_ of the HY strain is 100 or more times greater than the Y_SM_ of its parental WT strain, that is, x_HY_ ≪ x_WT_ [43,49,257,310].

It was shown that an increase in the dose of gamma rays led to a consistent increase in the production of PKS-based pigment melanin in *Cryomyces antarcticus* [311]. Exposure to ultraviolet irradiation led to an increase in pigmentation in both the melanin-containing *Cladosporium cladosporioides* and the non-melanized fungus *Paecilomyces variotii* [312]. However, in these cases, it is important to correlate how strongly the changes in Y_SM_ occurred in response to different doses of SP. Depending on this, the “x” parameter can increase, decrease, or remain unchanged.

### 7.2. Extension of Piano Model for Describing the Principle of SM Production—Organ Model

**Figure 7:**
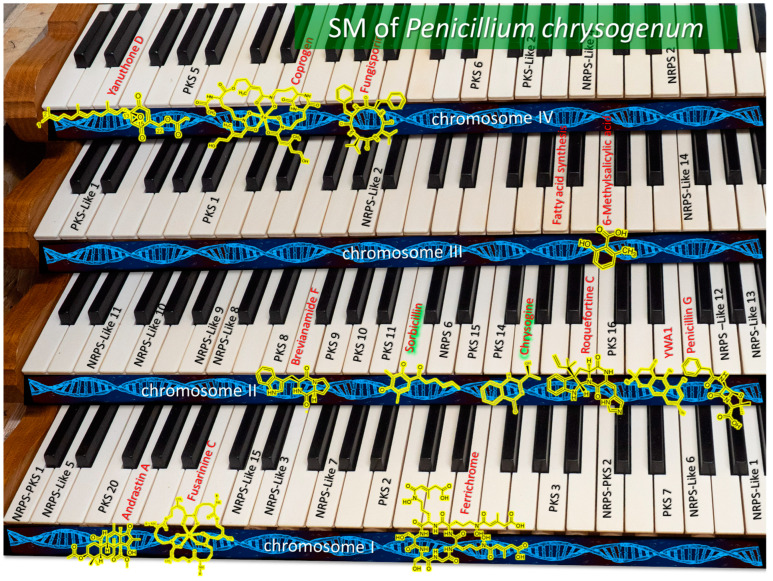
Localization of biosynthetic gene clusters (BGCs) for the production of secondary metabolites (SMs) on the chromosomes of *Penicillium chrysogenum*. Four chromosomes of *P. chrysogenum* are compared with four keyboards of the organ; the relative localization of BGCs on keys of chromosome keyboards is shown, as previously described [51]. BGCs for which the product is unknown (“orphan” clusters) are designated according to the previously entered numbering [51] and are marked in black. The BGC for which the product is known is indicated by its name and marked in red; the formula given for this target metabolite is marked in yellow.

The model in Figure 6 simplifies the organization of BGCs observed in the fungal cell (which are mosaically arranged along chromosomes), since the depicted musical instrument (piano) has only one keyboard, on which BGC keys are conventionally placed. However, fungi have from two to several dozen chromosomes. Therefore, for a more illustrative example of the distribution of BGCs over a genome, an organ with several keyboards, each of which corresponds to one chromosome of the fungus, can be used. The transition to the organ model also makes it possible to map the BGC loci mosaically located in the chromosomes of the fungus genome to the keys corresponding to the keyboard chromosomes. As an example of such visualization, the model organism *P. chrysogenum* was used, for which gene clusters were studied in detail and mapped onto chromosomes [51], and which has only four chromosomes; therefore, the corresponding organ has four keyboards (Figure 7). This is easier to visualize as another fungal model organism with relatively well studied and mapped BGCs, *Aspergillus nidulans*, has eight chromosomes [36]; hence, the “organ” for *A. nidulans* has eight keyboards. The BGCs with known secondary metabolites for *P. chrysogenum* are marked in red, and next to them are the structural formulas of the produced SMs. Orphan BGCs are marked in black (Figure 7).

Recent studies show the complex topological organization of chromosomes in the nucleus of a fungal cell [313]. It is also still unclear how the system of regulation of secondary metabolism manages epigenetic status in BGC loci mosaically scattered over chromosomes. Perhaps these loci are spatially close in the nucleus, which ensures their joint regulation, or there are other “threads” that allow the regulatory system puppeteer to pull them at the right time.

### 7.3. Improvement of Fungal Strains for Target SM Production in Light of the Piano Model

In order to create an HY producer from a WT strain, it is necessary to overcome the regulation at some built-in levels. A natural isolate has coordinated signal regulation (regulation in response to a signal), but for an industrial producer, it is necessary that synthesis of the target SM always occurs and is not mediated by the signal. Therefore, it is necessary to break down (overcome) barriers to increased production. It turns out that the selection of high-yielding clones after random mutagenesis works in this way; it works against systems that restrict the overproduction of SMs of interest [49]. Selection, first of all, destroys the protective locks that do not allow the dam to break, to eliminate the entire possibility of the cell’s biosynthetic potential for the production of only one type of molecule, which it does not need in such quantities. But this molecule is necessary for humans, in the biotechnological industry, and we artificially force the cell to perform functions that it is not adapted for in the environment; we convert a normal ecologically complete organism into a cellular factory intended for one and only one biosynthetic program.

First, it is necessary to remove the epigenetic control of secondary metabolism biosynthesis, which “holds” unclaimed BGCs, in terms of their signal, in the heterochromatin status. Mutations in the system of global regulation of secondary metabolism were seen as one of the most significant in an analysis of molecular changes in an SCI program for *P. chrysogenum* DS17690 [49]. In particular, the authors paid attention to mutations in the velvet complex such as: (i) 315 Gln → Stop in *velA* (leading to a decrease in the affinity between VelA and LaeA) and (ii) 338 Gly → Ser and 248 Lys → Glu in *laeA*.

In addition, it is most likely that it is the epigenetic control of BGCs that, in many cases, leads to the “silence” of duplicated copies of the target BGC. In other words, if as a result of the SCI program, there was no escape from the epigenetic system of global regulation, then, probably, in the case of duplications, only one of the target BGCs would be expressed. Other copies would be in heterochromatin status. Thus, a detailed study of the industrial strain *P. chrysogenum* P2niaD18, which, after the CSI program, had two copies of the target BGC, showed that the production of PenG is not dependent on the copy number of biosynthesis genes [314]. And the extraordinary high level of PenG production in *P. chrysogenum* DS17690 (with eight copies of the target BGC), among other changes, may be due to detected changes in the velvet system, which could allow more than one copy of the beta-lactam gene cluster to be expressed [49].

Since epigenetic control of the regulation of secondary metabolism is global, the influence on this chromatin-remodeling system can also lead to the activation of alternative BGCs to the target BGCs. This is probably why, along with mutations in the system of regulation of secondary metabolism, CSI programs select mutations that lead to the inactivation of alternative BGCs.

The active production of alternative SMs is highly undesirable for industrial production, since the cell spends additional resources on their biosynthesis, which could be redirected to obtain the target compound. In addition, their admixture complicates the purification process. Therefore, in those clones in which mutations occurred in the key genes of alternative BGCs during CSI, the production of the target BGC could increase, which serves as a selection factor. Secondly, the selection also takes into account the presence of impurity compounds. Other things being equal, clones with a smaller amount of impurity products are selected for further use, which facilitates purification. As a result, during selection, clones with disrupted production of one actively expressed alternative SM or another receive an advantage. For example, in *P. chrysogenum* DS17690 during CSI, 8 out of 31 studied megasynthase genes were targeted, with a corresponding and progressive loss in the production of a range of SMs unrelated to β–lactam production [49]. And a promising approach associated with the development of universal fungal recipient strains for the heterologous expression of BGCs was based on the modification of HY strains through inactivation of the most active host BGCs [58].

From the point of view of the piano model, in order to create an industrial HY strain, it is necessary to greatly modify a perfectly created piano for the finely tuned regulation of SMs in natural conditions (the extraction of various sounds) (Figure 7). It is required to eliminate the control of the signal-dependent regulatory system, since it is not desirable for an industrial strain to depend on any environmental signals. First of all, it is necessary to break the fallboard and keyboard cover (make mutations in the secondary metabolism regulation system at the epigenetic level). But then, chaos can arise, a cacophony in the production of SMs in the cell. This has been demonstrated in a series of works on genetic engineering manipulations with the global regulators of secondary metabolism, such as VelA or LaeA, which led to extremely serious changes in the entire profiles of secondary metabolites of the cell [57,315]. In particular, the overexpression of *laeA* in *Aspergillus niger* FGSC A1279 resulted in the upregulation of 281 putative secondary metabolite genes, including 22 backbone genes of BGCs [315]. The deletion of *velA* in the CSI-strains *P. chrysogenum* P2niaD18 and *A. chrysogenum* A3/2 led to a change in expression in both strains of approximately 50% secondary metabolite cluster genes, including β-lactam biosynthesis genes [57]. Without a regulation system, all sounds begin to sound simultaneously, while an HY strain should have only one sound, emitted by pressing the necessary key for the production of the required SM. Therefore, it is also necessary to break all the other keys of the keyboard, so that only one key remains, emitting the sound that is correct from the point of view of industrial fermentation. And this is exactly how (against the biosynthesis of alternative SMs) selection is directed in CSI programs, since one of the most important types of mutations selected during classical improvement turns out to be deletions in the backbone genes of alternative BGCs.

In addition to epigenetic control and transcription factors, the work of BGCs is obviously affected by some low-molecular-weight compounds through a mediating effect on the regulators and enzymes of the biosynthesis of secondary metabolism. One notable example is aliphatic polyamines, which upregulate *laeA*, target biosynthetic genes, and the production of target SMs in the CSI-strains *P. chrysogenum* Wisconsin 54-1255 and *A. terreus* 43-16 [44,301]. Such an effect can be associated with pressing the forte pedal (right pedal), which serves to lengthen the sounds and raises all dampers at once, so that after the key is released, the corresponding strings continue to sound. One explanation for their effects is related to the consumption of a common substrate, S-adenosine methionine (SAMe), which is required both for aminopropylation in the biosynthesis of polyamines and for the work of LaeA, a histone methylase. Using the example of *A. chrysogenum* RNCM 408D, it was shown that during a CSI program, the content of polyamines in a cell can be significantly increased [316]. This could be due to concomitant mutations selected at the sublethal level, since polyamines are able to protect the cell from peroxide stress and participate in the repair of DNA breaks in combination with RAD51 recombinase [317,318,319]. And the addition of exogenous polyamines during the fermentation process via a feedback mechanism inhibits their endogenous synthesis, which releases part of SAMe for the purpose of methylation and the work of LaeA. However, in specially designed genetically engineered strains or on specially selected nutrient media, the addition of polyamines can lead to a decrease in target production [169,244]. Such an effect can be associated with pressing the left pedal, which is used to dampen the sound.

## 8. Conclusions

Biosynthetic gene clusters (BGCs) of secondary metabolites (SMs) play a key role in their production. Inside a fungal cell is a “keyboard” of several dozen keys—BGCs—mosaically scattered across the chromosomes. A special signal-dependent hierarchical regulatory system “presses” the keys of this keyboard to express the necessary BGCs and produce the appropriate SMs. The system as a whole works on the principle of a piano; when a specific key (BGC) is pressed, the corresponding sound appears (the corresponding SM is synthesized). In order to turn a natural isolate into a high-yielding industrial producer, it is necessary to significantly change the natural instrument, leaving only one loud and constantly sounding key.

The main method by which commercial fungal producers have been obtained for decades is classical strain improvement (CSI), based on random mutagenesis and screening. In recent years, due to multiomics approaches, it has become clear what changes occur at the molecular level in fungal cells during such improvements. In an industrial fungal producer, key streams of primary metabolism are redirected, and the physiology, morphology, and life cycle of the strain can be significantly changed to adapt to specific fermentation parameters. However, the key factor is the upregulation of biosynthetic genes against the background of changes in the system of regulation and disruption of alternative BGCs.

## Figures and Tables

**Table 1:** Examples of the main types of fungal secondary metabolites: major producers, chemical properties, and biological action.

Secondary Metabolite	Producer	M	Chemical Structures	Effect	Type	References
Penicillin G	*Penicillium* *chrysogenum*	334	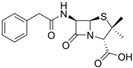	Antibiotic (penicillin, beta-lactams)	NRPS	[2,41]
Cephalosporin C	*Acremonium chrysogenum*	415	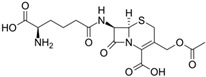	Antibiotic (cephalosporins, beta-lactams), feedstock for production of cephalosporins	NRPS	[69,70]
Enniatin A	*Fusarium* sp.	682	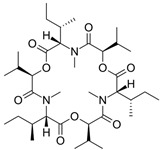	Antitumor	NRPS	[71]
Ergotamine (Ergomar^®^, TerSera Therapeutics LLC, Lake Forest, IL, USA)	*Claviceps* *purpurea*	582	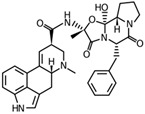	Induces vasoconstriction (acute migraine treatment)	NRPS	[72]
Fungisporin	*P. chrysogenum*	493	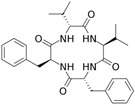	Antibiotic	NRPS	[73]
Patulin	*Aspergillus*, *Penicillium*, *Byssochlamys*	154	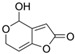	Antibiotic (discontinued due to high toxicity)	PKS	[74]
Lovastatin (Mevacor^®^, Merck Research Laboratories, Rahway, NJ, USA)	*Aspergillus terrius*	405	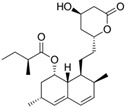	Cholesterol-lowering drug	PKS	[75,76]
Compactin (Mevastatin)	*Penicillium* *citrinum*	391	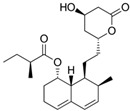	Cholesterol-lowering feedstock for production of Pravastatin^®^	PKS	[77,78]
Citrinin	*P. citrinum*	250	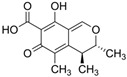	Mycotoxin with antibiotic activity	PKS	[79,80]
Griseofulvin	*Penicillium* *griseofulvum*	353	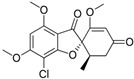	Antibiotic	PKS	[81]
Afidicolin	*Akanthomyces muscarius*	338	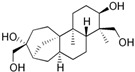	Antitumor (under testing)	Terpenoid	[82]
Fusidic acid (Fucidin^®^, Boehringer Ingelheim, Ingelheim, Germany)	*Fusidium* *coccineum*	517	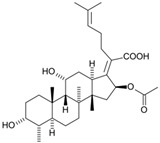	Antibiotic (fusidane-type antibiotic)	Terpenoid	[83,84]
Helvolic acid (Fumigacin)	*Aspergillus* *fumigatus*	569	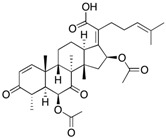	Mitotoxin with antibiotic activity (fusidane-type antibiotic)	Terpenoid	[85,86]
Paclitaxel (Taxol^®^)	*Aspergillus* *fumigatus*	854	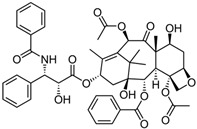	Antitumor (the most-used natural anticancer drug)	Terpenoid	[87,88]
Cephalosporin P1	*A. chrysogenum*	575	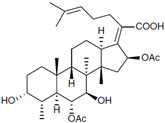	Antibiotic (fusidane-type antibiotic)	Terpenoid	[89,90]
Aspernigerin	*Aspergillus niger*	433	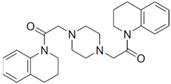	Cytotoxic (vs. tumor cell lines)	Alkaloid	[91,92]
Camptothecin	*P.chrysogenum* *A. terreus* *Alternaria* sp.	348	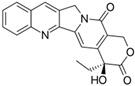	Antitumor (topoisomerase I inhibitor)	Alkaloid	[92,93,94,95]
Penicidone B	*Penicillium* sp.	387	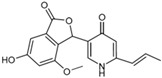	Antitumor	Alkaloid	[92,96]
Chaetominine	*Chaetomium* sp.	402	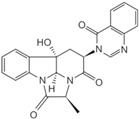	Antitumor	Alkaloid	[92,97,98]
Pneumocandin B_0_	*Glarea lozoyensis*	1065	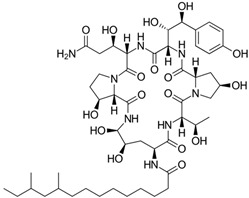	Antibiotic (echinocandins, lipopeptides), feedstock for production of caspofungin (Cancidas^®^)	NRPS/PKS	[99,100]
Echinocandin B	*Aspergillus* *nidulans*	1060	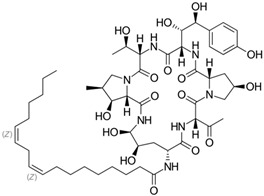	Antibiotic (echinocandins, lipopeptides), feedstock for production of anidulafungin (Eraxis^®^, Pfizer Medical Information, New York, NY, USA)	NRPS/PKS	[99,101]
Fusarin C	*Fusarium* sp.		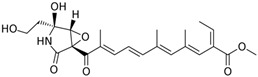	Mycotoxin, potent mutagen (affects agricultural crops)	NRPS/PKS	[102]
Chaetoglobosin A	*Penicillium* *expansum*	529	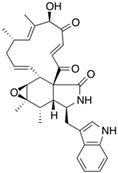	Antitumor (binds to actin filaments)	NRPS-PKS	[102,103,104]
Cyclosporin A	*Tolypocladium* *inflatum*	1203	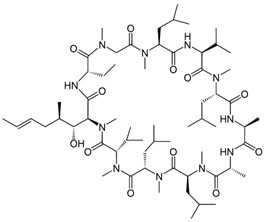	Immunosuppressant (agonist of immunophilin)	NRPS/PKS	[105]
Aculene A	*Aspergillus* *aculeatus*	315	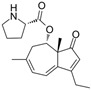	Function unknown	NRPS/terpenoid	[67,106,107]
Proversilin C	*Aspergillus* *versicolor*	439	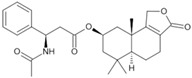	Antitumor	NRPS/terpenoid	[67,108]
Ergokonin A	*Trichoderma* sp.	544	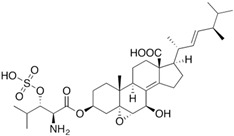	Antifungal activity	NRPS/terpenoid	[67,109]
Flavunoidine	*Aspergillus* *nidulans*	503	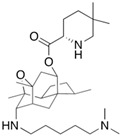	Function unknown	NRPS/terpenoid	[67,110]
Chevalone E	*Aspergillus* *similanensis*	415	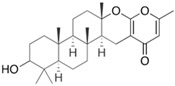	Antimicrobial	PKS/terpenoid	[67,111,112]
Viridicatumtoxin A	*Penicillium* *viridicatum*	566	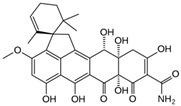	Antibiotic (tetracycline-like)	PKS/terpenoid	[81,113]
Arthripenoid A	*Arthrinium* sp.	592	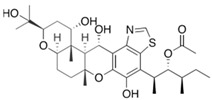	Antimicrobial, cytotoxic, immunosuppressive	PKS/terpenoid	[67,114]
Fumagillin	*Aspergillus* *fumigatus*	459	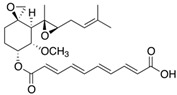	Antibiotic (polyenes), mycotoxin	PKS/terpenoid	[115,116,117,118,119]
Terreuspyridine	*Aspergillus terreus*	542	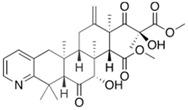	Inhibitory effects (vs. butyrylcholinesterase)	Alkaloid/ terpenoid	[67,120]
15-deoxyoxalicine B	*Penicillium* *oxalicum*	488	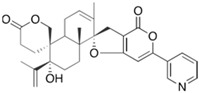	Function unknown	Alkaloid/ terpenoid	[67,121]
Chartarlactam A	*Stachybotrys chartarum*	400	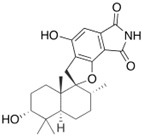	Antihyperlipidemic	Alkaloid/ terpenoid	[122]
Dysivillosin A	*Dysidea* *villosa*	408	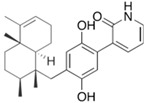	Anticleric	Alkaloid/ terpenoid	[123]
Biscognienyne b	*Biscogniauxia* sp.	260	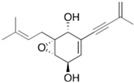	Antitumor	Other (MUS)	[67,124,125]
Alternarin A	*Alternaria* sp.	494	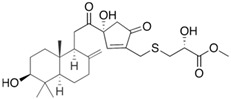	Antiepileptic (inhibitory activity on neuronal excitability)	Other (MUS)	[126]
(R,R)-Rhizoferrin	*Rhizopus* *delemar*	436	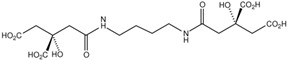	Siderophore (carboxylate type)	Other (NIS)	[127]
Cerulenin	*Cephalosporium caerulens*	569	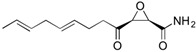	Antibiotic, also has an antitumor effect	Other (related with fatty acid synthesis)	[128,129,130]
Kojic acid	*Aspergillus oryzae*	142	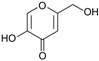	Antimicrobial (used in pharmaceuticals, cosmetics, and food industry)	Other	[131,132,133]

M—molecular mass.

## Data Availability

The data are contained within the article.
